# Intra- and Inter-Rater Reliability and Validation of Orofacial Cleft Classification in the Cleft Collective Cohort Study

**DOI:** 10.1177/10556656261443367

**Published:** 2026-05-10

**Authors:** Amy Davies, David Sainsbury, The Cleft Multidisciplinary Collaborative, Jibby Medina, Craig Russell, Kerry Humphries, Kate J. Fitzsimons, Jonathan Sandy, Scott Deacon, Yvonne Wren

**Affiliations:** 1 The Cleft Collective, 1980 Bristol Dental School, The University of Bristol, Bristol, UK; 2 Bristol Dental School, The University of Bristol, Bristol, UK; 3 105563 Cleft Lip and Palate service, Newcastle upon Tyne Hospitals, Newcastle upon Tyne, UK; 4 Cleft Multidisciplinary Collaborative, London, UK; 5 Cleft Registry and Audit NEtwork (CRANE) Database, Clinical Effectiveness Unit, 14211 Royal College of Surgeons of England, London, UK; 6 Cleft Registry and Audit NEtwork (CRANE) Database, Clinical Effectiveness Unit (CEU), Royal College of Surgeons of England and National Cleft Surgical Service For Scotland, 59842 Royal Hospital for Children, Glasgow, UK; 7 1984 University Hospitals Bristol and Weston NHS Foundation Trust, Bristol, UK; 8 Cardiff School of Sport & Health Sciences, 11352 Cardiff Metropolitan University, Cardiff, UK; 9 Bristol Speech & Language Therapy Research Unit (BSLTRU), North Bristol NHS Trust, Bristol, UK

**Keywords:** The Cleft Collective, CRANE, Cleft Multidisciplinary Collaborative, UK cleft care, cleft classification, cleft lip, cleft palate, cleft lip and palate

## Abstract

**Objective:**

Assess the level of agreement for classification of orofacial clefts within and between different raters and validate classifications.

**Design:**

Validation study.

**Setting:**

National longitudinal prospective cohort, United Kingdom.

**Participants:**

Children born with orofacial cleft (n=4211), recruited to the Cleft Collective between 2013 and 2024.

**Main outcome measures:**

Four cleft classifications of orofacial clefts were explored. Classification one comprised cleft lip, cleft palate, and cleft lip and palate. Classification two added laterality, classification three added sidedness and classification four added completeness using LAHSAL. Data on orofacial cleft classification were collected through seven different sources.

**Results:**

At least one report of cleft classification was available for 4052 Cleft Collective study children. When assessing intra-rater agreement mothers had the highest level of agreement for the simplest form of cleft classification with a Krippendorf's Alpha of 0.987. When recording LAHSAL for the same child, surgeons reported the same classification for 71% of children (Krippendorf's Alpha = 0.672). When assessing inter-rater agreement across different sources, the simplest cleft classification resulted in the highest level of agreement (Krippendorf's Alpha = 0.957) and the least agreement when using LAHSAL (Krippendorf's Alpha = 0.538).

**Conclusion:**

Our study found that the more complex a cleft classification becomes the less agreement there is between sources. Differences across sources became most apparent when reporting the sidedness or completeness of a cleft. Validation of cleft classification is important for both clinical care and research. Although LAHSAL is advocated for use both clinically and in research our data show that rigorous training is essential.

## Introduction

Orofacial clefts present clinically with different phenotypes.^1,2^ There are various classifications for these orofacial cleft phenotypes but no consensus on which classification system should be used universally.^3,4^ The simplest classification identifies the oral structure in which the cleft occurs: cleft lip, cleft palate, or cleft lip and palate. Cleft lip may occur unilaterally or bilaterally and if unilateral it can be classified as left or right. More detailed classifications which include information on the extent of the cleft are available^3^; For example, Krein's LAHSHAL classification was introduced in 1989^5^ where the first letter represents the anatomy where a cleft may occur, from the right lip through to the left lip, detailing the involvement of the alveolus, hard palate and soft palate.^5,6^ Each letter within the LAHSHAL palindrome can be capitalised to represent a complete cleft or in lower case to represent an incomplete cleft. Where a cleft is not present the letter is replaced with a period or dot.^4^ Further, an asterisk and/or plus sign may be included to denote a less substantial cleft in any location (ie, microform, notch or submucous) or the presence of Simonart's bands of the lip, respectively.^4^ In 1995, the Royal College of Surgeons of England, recommended shortening and simplifying LAHSHAL to LAHSAL where the hard palate is represented once rather than bilaterally.^7^

The LAHSHAL and LAHSAL classification are widely used and recommended.^4,8,9^ Unlike some other cleft classifications, the use of LAHSHAL is complementary to the internationally recognised International Classification of Diseases 10^th^ and 11^th^ revisions (ICD-10 and ICD-11, respectively) systems and easy to record on electronic records.^10,11^ McBride et al. reported excellent reliability and accuracy in the use of the LAHSAL classification in the electronic records for the Cleft Service in Scotland (CleftSiS).^8^ This further supports the use of LAHSAL more widely. Houkes et al. highlighted the advantages of LASHAL but found considerable disagreement between raters when classifying an orofacial cleft before receiving training on the LAHSHAL classification system. The inter-rater reliability improved following training through a webinar, although inconsistencies in the classification across raters were still present post training.^12^ Wang et al. found that nonspecialist healthcare providers were more accurate using a cleft classification system named RPL (Right-Palate-Left^13^) compared to LAHSHAL coding.^14^ They concluded that LAHSHAL was complex for nonspecialist healthcare providers but acknowledged the benefits of the detailed anatomical description of LAHSHAL for both clinical care and research.^14^

Detailing the phenotype of an orofacial cleft accurately is important for both clinical care and epidemiological studies.^2^ The type of cleft determines the treatment needed and likely outcome as well as what information is needed by the patient and the family.^15–18^ Moreover, cleft phenotypes have differing aetiologies and outcomes.^1,2,19,20^ Differences in aetiology and outcomes occur, not only between the simplest definition of phenotype (cleft lip, cleft palate or cleft lip and palate), but also between the laterality and sidedness of a cleft lip (with or without palate) and the completeness of the cleft.^21,22^ To fully understand the extent of these differences between cleft phenotypes, accuracy in the recording of cleft classification and rich longitudinal data are needed.^23^

The Cleft Collective is a longitudinal cohort study of children born with an orofacial cleft and their families. The Cleft Collective recruits participants from across the whole of the United Kingdom (UK) from each of 16 centralised cleft centres.^24^ Data on the child's phenotype, maternal and paternal environmental factors, biological samples, functional and clinical data, and parental and self-reported data are collected throughout the development of the study child. The resource is available to clinicians and researchers worldwide and provides an opportunity for collaborators to access rich data whilst reducing the burden of recruiting participants and collecting data themselves. Details on how to access the resource can be found at https://www.bristol.ac.uk/cleft-collective/professionals/access/.

Given the challenges inherent in cleft classification and the importance of accurate sub-phenotyping of an orofacial cleft we investigated the classification of cleft for participants within the Cleft Collective. The aim of our study was to assess the level of agreement for cleft classification within and between different raters and to validate cleft classification within the Cleft Collective. The following three objectives were undertaken to address this aim; 1) compare internal consistency of cleft classification, 2) compare external agreement of cleft classification, and 3) derive a validated cleft classification for use within the Cleft Collective resource.

## Methods

### Governance

Ethical approval for the Cleft Collective was obtained from the South West NRES (REC 13/SW/0064). At the point of recruitment, participants of the Cleft Collective were asked to provide optional explicit consent for the Cleft Collective to be able to link to their medical records and/or the cleft registry and audit network (CRANE) database. Data sharing between the Cleft Collective and the CRANE Database was outlined within a data sharing agreement. This study was conducted under project number CC021-AD.

### Data Collection

Data on cleft classification for the study children within the Cleft Collective were obtained from seven different sources. Five sources of data were collected by the Cleft Collective and two were collected externally and then linked to the Cleft Collective resource. Details of all seven sources are outlined below.

#### The Cleft Collective

*Sources 1 and 2:* Throughout the study child's development, mothers (maternal questionnaires) and fathers or mother's partners (paternal questionnaires) who have consented to the Cleft Collective are asked to complete questionnaires at key timepoints.^25^ Data from questionnaires collected at the time of recruitment and when the child was 18 months, 3 years, 5 years, 8 years and 10 years were used within this study. Within each of these questionnaires the parent/guardian were asked to classify their child's cleft by answering three questions. Initially parents/guardians were asked whether their child was born with a cleft lip, cleft palate or a cleft lip and palate. Where a child was born with a cleft involving the lip, the parent/guardian was further asked to specify the laterality of the cleft (unilateral or bilateral). Finally, where a child was born with a unilateral cleft lip, with or without cleft palate, the parent/guardian was asked, when looking at their child, does the cleft appear on the left or right side, hereafter the side in which a unilateral cleft lip presents is described as ‘sidedness’. When coding the sidedness of the cleft, left was swapped to right and vice-versa to ensure the sidedness related to the child rather than the reflective image that would have been seen by the parent/guardian. Within this study, responses to the maternal and paternal questionnaires were initially explored independently but were then combined (parental questionnaires).

*Source 3:* Biological samples are obtained from Cleft Collective participants at the point of recruitment.^24^ Samples are sent to the Bristol Bioresource Laboratory, UK, and are accompanied by a biological sample transfer form. In 2016, the Cleft Collective introduced three additional questions to the biological sample form to detail the study child's cleft classification. These questions are the same questions asked within the parental questionnaires, detailing the affected orofacial structures, laterality and sidedness of the child's cleft. Biological sample transfer forms are completed by the person obtaining the sample, this could be a member of the cleft team or the parents/guardians.

*Source 4:* For study children who are recruited to the Cleft Collective before their primary cleft repair, a short surgical form detailing the child's surgery is requested from the cleft team. The short surgical form is a questionnaire asking about the techniques and adjuncts used during surgery, the child's medical history and cleft phenotype. Cleft phenotype includes the classification of the child's cleft recorded as LAHSHAL or LAHSAL. The completion of short surgical forms is requested at each of the child's cleft related surgeries, resulting in the possibility of multiple forms being obtained for one study child.

*Source 5:* At the point of recruitment, parents/guardians are asked to provide consent for the Cleft Collective to obtain data from the child's medical records held by the cleft team. Approximately 96% of participants provided consent for this optional data linkage. Between 2018 and 2020, authors AD and JS visited ten cleft centres from across the UK to obtain data about the study children directly from medical records. Data included cleft classification, recorded as LAHSHAL or LAHSAL. For a small minority, cleft classification differed within the medical records held for the individual study child. Where discrepancies were apparent within a child's medical record, the classification was checked against pre-operative photographs or surgical diagrams. Where photographs or surgical diagrams were not available the most frequently occurring classification was recorded for the study child.

#### The Cleft Multidisciplinary Collaborative (CMC)

*Source 6:* The CMC was established in 2016 as a multidisciplinary trainee collaborative within the field of cleft. The aim of the CMC was to help improve clinical practice in cleft care by undertaking collaborative projects such as audits and research.^26^ One of the first projects the CMC embarked on was to help provide cleft classifications for study children where data were not already available within the Cleft Collective resource. Members of the CMC were provided with lists of Cleft Collective participants within their cleft centre who had consented to the Cleft Collective accessing medical data held by the cleft teams. Lists provided to CMC members were encrypted to the Advanced Encryption Standard 256 (AES-256) and were password protected before being securely transferred. Pseudonymous data about the study participants, including cleft classification in the form of LAHSHAL and LAHSAL, were collected via Jisc online surveys using study identification (ID) numbers. Data were collected by 18 trainees from a total of ten UK cleft centres.

#### Cleft Registry and Audit NEtwork (CRANE)

*Source 7:* The CRANE Database is a national clinical registry and audit collecting information about all children born with a cleft lip and/or palate in the United Kingdom of Great Britain and Northern Ireland. The database was established by the Department of Health in 2000.^27^ Data collection from Scotland commenced in 2023.^28,29^ A one-off linkage between the Cleft Collective and the CRANE Database was undertaken in 2020. Linkage ID numbers for each individual study child were derived to ensure Cleft Collective and CRANE IDs were not shared between sources. Linking variables included the child's full name, date of birth and recruitment site. Data from study children recruited in Scotland were not available within the CRANE Database at the time of linkage. Linking data from the Cleft Collective was encrypted to AES-256 and sent to the CRANE Database team to perform the linkage between the two data sources. Linkage was conducted using the statistical software package Stata using a Stata do-file derived by authors AD, JM and KF. Following linkage, data were once again encrypted to AES-256 and sent from CRANE to the Cleft Collective. Cleft classification was obtained from CRANE in the form of LAHSAL.

### Cleft Classifications

To enable comparison of cleft classification across sources, all data were coded into three different cleft classifications, each constructed to match detail available. A fourth cleft classification was utilised for sources where data were provided as either LAHSAL or LAHSHAL. The first cleft classification (CC1) to be compared was the least detailed, denoting only the affected orofacial structures. Categories for CC1 comprised: cleft lip; cleft palate; cleft lip and palate. The second cleft classification (CC2) expanded on CC1 to include the laterality of the cleft. Categories for CC2 comprised: unilateral cleft lip; bilateral cleft lip; cleft palate; unilateral cleft lip and palate; bilateral cleft lip and palate. The third cleft classification (CC3) further expanded on CC1 and CC2 by including the sidedness of the unilateral cleft lip with or without palate. Categories for CC3 comprised: right sided unilateral cleft lip; left sided unilateral cleft lip; bilateral cleft lip; cleft palate; right sided unilateral cleft lip and palate; left sided unilateral cleft lip and palate; bilateral cleft lip and palate. The fourth classification to be explored, where available, was LAHSAL. Where data were available for LAHSHAL, these data were converted into the simplified LAHSAL and subsequently into the categorical variables CC1, CC2 and CC3. The acronyms CC1, CC2 and CC3, have been used for the ease of reporting for the purpose of this analysis and are not considered official terminology.

Data on CC1, CC2, CC3 and LAHSAL were compared. Study children who presented with a different cleft type than that outlined above, such as a submucous cleft palate or a microform cleft lip, were excluded from this study.

### Statistical Analyses

Intra-rater agreement was assessed for maternal and paternal responses to parental questionnaires. Where a mother or father (or mother's partner) had reported their child's cleft classification at multiple timepoints, the percentage of parents who had perfect agreement across the timepoints was reported. Due to the nominal nature of the data and the mixed number of responses, Krippendorf's alpha was used to measure the level of agreement between the same responders for cleft classifications CC1, CC2 and CC3 provided at multiple timepoints. A final maternal and paternal response to each cleft classification available were then derived using either the most reported classification over time or the single, one-time response.

Intra-rater agreement was further assessed within cleft classification reported within Cleft Collective short surgical forms. Where the same surgeon had completed multiple forms, reported cleft classifications were compared across timepoints. Overall percentage of agreement between the same responders was reported and Krippendorf's alpha was calculated for all possible cleft classifications. A final surgical response to each cleft classification available was then derived using either the most reported classification over time or the single, one-time response.

Inter-rater agreement was assessed between the final maternal and paternal responses derived from the earlier intra-rater analyses for cleft classifications CC1, CC2 and CC3. The overall percentage of agreement between maternal and parental report and Krippendorf's alpha were calculated for each classification. A final parental cleft classification was determined by one of the following scenarios: 1) perfect agreement between parental report of cleft classification, 2) most frequently reported cleft classification between parental report, or 3) the final maternal or paternal classification where only one parent had provided a response.

Percentage of agreement and Krippendorf's alpha were calculated for all the different combinations of two sources across all cleft classifications, CC1, CC2, CC3 and LAHSAL. Matrices were derived to visually display these results. The percentage of overall agreement between all sources available was reported for each of the cleft classifications. Krippendorf's alpha was calculated to further assess agreement and inter-rater agreement between sources.

A final cleft classification for each child was determined by one of the following scenarios: 1) perfect agreement between all sources, 2) most frequently reported cleft classification across sources, or 3) only one response across all sources. Scenarios 1 and 2 were considered validated cleft classification. Cleft classification was not considered to be validated where there was only a single response across all sources or where there was unresolvable disagreement across sources. The proportion of validated cleft classifications across all classifications explored was described.

All calculations of Krippendorf's Alpha incorporated bootstrapping with 1000 iterations to derive a bootstrap sampling distribution; in turn this enabled the calculation of 95% confidence intervals (95%CIs) as described by Hayes and Krippendorf.^30^ Krippendorf's Alpha calculates a value between −1 and +1, where a Krippendorf's Alpha value of +1 suggests perfect agreement, a value of zero suggests no agreement other than what may be seen by chance and a value less than zero suggests systematic disagreement.^31,32^ Krippendorf's Alpha values can be interpreted as a satisfactory level of agreement for values greater than or equal to 0.80, moderate agreement for values between 0.67 and 0.79 and values less than 0.67 should be interpreted as inconsistent coding between raters.^31,32^

Data were cleaned and analysed using Stata/MP version 18^33^ and Krippendorf's alpha was calculated using the K-Alpha calculator.^32^

## Results

### Sample

At the time of analyses, the Cleft Collective comprised 4211 study children within the cohort, recruited between November 2013 and December 2024. Parental report on cleft classification were available for 2486 children from at least one parental questionnaire, 2079 children from surgical forms, 2477 children from biological sample transfer forms, 1061 children from medical records, 787 children from the CMC and 2176 children from CRANE, these numbers are not mutually exclusive. At least one report of cleft classification, from any source, was available for 4052 (96.2%) children.

### Agreement Within Sources: Intra-Rater Agreement

#### The Cleft Collective Parental Questionnaires

At least one maternal response on cleft classifications CC1, CC2 and CC3 was available for 2355, 2335 and 2325 children, respectively. Of these children, more than one maternal report on cleft classifications CC1, CC2 and CC3 were available for 1437, 1409 and 1393 children, respectively. Where multiple reports were available for CC1, 98.8% (n = 1420) of mothers reported the same cleft type at each timepoint for their child resulting in a Krippendorf's Alpha value of 0.987 (95% CIs 0.98, 0.99), suggesting almost perfect agreement. For CC2, 97.6% (n = 1375) of mothers reported the same cleft type at each timepoint, Krippendorf's Alpha was 0.975 (95% CIs 0.97, 0.98). For CC3, 80.5% (n = 1121) of mothers reported the same cleft type at each timepoint, Krippendorf's Alpha was 0.808 (95% CIs 0.79, 0.83), suggesting satisfactory agreement. From the original maternal sample, a final maternal cleft classification was determined for 99.5% (n = 2343) for CC1, 99.3% (n = 2318) for CC2 and 94.5% (n = 2197) for CC3.

At least one paternal response on cleft classifications CC1, CC2 and CC3 were available for 1569, 1554 and 1546 children, respectively. Of these children, more than one paternal report on cleft classifications CC1, CC2 and CC3 were available for 784, 762 and 754 children, respectively. Where multiple reports were available for CC1, 98.3% (n = 771) of fathers or mother's partners reported the same cleft type at each timepoint for their child resulting in a Krippendorf's Alpha value of 0.980 (95% CIs 0.97, 0.99). For CC2, 96.7% (n = 737) of fathers or mother's partners reported the same cleft type at each timepoint, Krippendorf's Alpha was 0.963 (95% CIs 0.95, 0.98). Both CC1 and CC2 had an almost perfect level of agreement. For CC3, 82.1% (n = 619) of fathers or mother's partners reported the same cleft type at each timepoint, Krippendorf's Alpha was 0.807 (95% CIs 0.78, 0.84), suggesting a satisfactory level of agreement. From the original paternal sample, a final paternal cleft classification was determined for 99.6% (n = 1563) for CC1, 99.1% (n = 1540) for CC2 and 94.7% (n = 1464) for CC3.

#### The Cleft Collective Short Surgical Forms

A LAHSAL cleft classification was available for 2079 children from the Cleft Collective short surgical forms. Of these children, a LAHSAL cleft classification was provided by the same surgeon, on up to four occasions, for 15.6% (n = 324) of the sample when reporting secondary surgeries. Where multiple reports for the same child by the same surgeon were available, 98.5% (n = 319) reported the same CC1 classification when converted from LAHSAL, with a Krippendorf's Alpha of 0.948 (95% CIs 0.90, 0.99). For CC2, 97.5% (n = 316) of surgeons reported the same cleft type at each timepoint, with a Krippendorf's Alpha of 0.963 (95% CIs 0.93, 0.99). For CC3, 93.8% (n = 304) of surgeons reported the same cleft type at each timepoint, with a Krippendorf's Alpha of 0.924 (95% CIs 0.89, 0.96). For LAHSAL, 71.0% (n = 230) of surgeons reported the same cleft type at each timepoint, with a Krippendorf's Alpha of 0.672 (95% CIs 0.62, 0.72). From the original short surgical form sample, a final surgical cleft classification was determined for 99.8% (n = 2075) for CC1, 99.7% (n = 2072) for CC2, 99.1% (n = 2060) for CC3 and 95.3% (n = 1986) for LAHSAL.

### Agreement Between Sources: Inter-Rater Agreement

#### The Cleft Collective Parental Questionnaires

At least one final cleft classification from either a maternal or paternal questionnaire was available for 2486 children for CC1, 2465 children for CC2 and 2395 children for CC3. When combined, both a final maternal and paternal cleft classification for the same child were available for 1420 children for CC1, 1393 children for CC2 and 1266 children for CC3. For CC1, final maternal and paternal cleft classification matched for 99.9% (n = 1419) of children, with a Krippendorf's Alpha value of 0.999 (95% CIs 0.997, 1.000) suggesting perfect agreement. For CC2, final maternal and paternal cleft classification matched for 99.6% (n = 1388) of children, with a Krippendorf's Alpha value of 0.995 (95% CIs 0.99, 1.00). For CC3, final maternal and paternal cleft classification matched for 93.9% (n = 1189) of children, with a Krippendorf's Alpha value of 0.918 (95% CIs 0.90, 0.94), suggesting a satisfactory level of agreement. From maternal and paternal questionnaires, a final parental cleft classification was determined for 99.96% (n = 2485) for CC1, 99.8% (n = 2460) for CC2 and 96.8% (n = 2318) for CC3.

#### All Sources

Data on classification CC1 from at least one source were available for 4052 children; data from at least two sources were available for 3183 children. Where data from two or more sources were available, cleft classification matched across the available sources for 96.0% (n = 3057) of the sample. This resulted in a Krippendorf's Alpha of 0.957 (95% CIs 0.95, 0.97), suggesting an almost perfect level of agreement between sources. The two sources with the least agreement were the Cleft Multidisciplinary Collaborative and Cleft Collective biological sample transfer forms, with a 94.9% match in CC1 and a Krippendorf's Alpha of 0.920 (95% CIs 0.85, 0.98), suggesting satisfactory agreement [Table 1]. The two sources with the most agreement were Cleft Collective parental questionnaires and medical records, with a 99.7% match in CC1 and a Krippendorf's Alpha of 0.996 (95% CIs 0.99, 1.00) suggesting an almost perfect agreement [Table 1]. A final CC1 cleft classification was determined for 4003 (98.8%) children, of which 78.3% (n = 3134) were validated.

**Table 1.** Matrix of Krippendorf's Alpha and Percentage Match for CC1 Cleft Classification Across Dual Sources.

Cleft Classification 1 (CC1) CL / CP / CLP	CC Parental Questionnaires	CC Biological Sample Transfer Forms	CC Surgical Forms	Medical Records	Cleft Multidisciplinary Collaborative	CRANE
CC Parental questionnaires		0.950 (0.93, 0.97)	0.984 (0.97, 0.99)	0.996 (0.99, 1.00)	0.978 (0.96, 0.99)	0.975 (0.96, 0.99)
CC biological sample transfer forms	96.8 (n = 839)		0.948 (0.93, 0.96)	0.967 (0.94, 0.98)	0.920 (0.85, 0.98)	0.935 (0.91, 0.95)
CC Surgical forms	98.4 (n = 841)	96.6 (n = 1466)		0.961 (0.94, 0.98)	0.964 (0.93, 0.99)	0.952 (0.94, 0.97)
Medical records	99.7 (n = 378)	97.8 (n = 449)	97.5 (n = 583)		0.961 (0.94, 0.98)	0.976 (0.96, 0.99)
Cleft Multidisciplinary Collaborative	98.6 (n = 276)	94.9 (n = 93)	97.7 (n = 291)	97.5 (n = 422)		0.962 (0.94, 0.98)
CRANE	98.4 (n = 736)	95.7 (n = 966)	96.9 (n = 961)	98.5 (n = 704)	97.6 (n = 559)	

*CC1 represents the simplest form of cleft classification where only the anatomical oral region of the cleft is specified: CL – Cleft Lip only; CP – Cleft Palate only; CLP – Cleft Lip and Palate.

**CC - Cleft Collective; CRANE - The Cleft Registry and Audit NEtwork (CRANE) database.

***Upper right side of matrix (blue) represents Krippendorf's Alpha from a nominal variable and 95% confidence intervals generated through bootstrapping with 1000 iterations (https://www.k-alpha.org/). Lower left side of matrix (orange) represents the percentage and number of children where an exact match between the two specified sources within the matrix was present.

Data on classification CC2 from at least one source were available for 4016 children, data from at least two sources were available for 3111 children. Where data from two or more sources were available, cleft classification matched across the available sources for 94.8% (n = 2949) of the sample. This resulted in a Krippendorf's Alpha of 0.949 (95% CIs 0.94, 0.96), suggesting a satisfactory level of agreement between sources. The two sources with the least agreement were the Cleft Multidisciplinary Collaborative and Cleft Collective Biological Sample Transfer Forms, with a 94.1% match in CC2 and a Krippendorf's Alpha of 0.909 (95% CIs 0.82, 0.98) suggesting satisfactory agreement [Table 2]. The two sources with the most agreement were Cleft Collective parental questionnaires and medical records, with a 99.5% match in CC2 and a Krippendorf's Alpha of 0.992 (95% CIs 0.98, 1.00) suggesting almost perfect agreement [Table 2]. A final CC2 cleft classification was determined for 3949 (98.3%) children and of those 77.1% (n = 3044) were validated across multiple sources.

**Table 2.** Matrix of Krippendorf's Alpha and Percentage Match for CC2 Cleft Classification Across Dual Sources.

Cleft Classification 2 UCL / BCL / CP / UCLP / BCLP	CC Parental Questionnaires	CC Biological Sample Transfer Forms	CC Surgical Forms	Medical Records	Cleft Multidisciplinary Collaborative	CRANE
CC Parental questionnaires		0.941 (0.92, 0.96)	0.975 (0.96, 0.99)	0.992 (0.98, 1.00)	0.980 (0.96, 1.00)	0.971 (0.96, 0.99)
CC biological sample transfer forms	95.8 (n = 758)		0.944 (0.93, 0.96)	0.951 (0.93, 0.97)	0.909 (0.82, 0.98)	0.919 (0.90, 0.94)
CC Surgical forms	98.2 (n = 817)	96.0 (n = 1334)		0.954 (0.93, 0.97)	0.957 (0.93, 0.98)	0.945 (0.93, 0.96)
Medical records	99.5 (n = 370)	96.5 (n = 415)	96.7 (n = 578)		0.948 (0.92, 0.97)	0.971 (0.96, 0.99)
Cleft Multidisciplinary Collaborative	98.6 (n = 273)	94.1 (n = 80)	97.0 (n = 288)	96.3 (n = 417)		0.947 (0.93, 0.97)
CRANE	98.0 (n = 718)	94.3 (n = 856)	96.0 (n = 951)	97.9 (n = 700)	96.2 (n = 551)	

*CC2 represents the anatomical oral region and laterality of the cleft: UCL – Unilateral Cleft Lip; BCL – Bilateral Cleft Lip; CP – Cleft Palate only; UCLP – Unilateral Cleft Lip and Palate; BCLP – Bilateral Cleft Lip and Palate.

**CC - Cleft Collective; CRANE - The Cleft Registry and Audit NEtwork (CRANE) database.

***Upper right side of matrix (blue) represents Krippendorf's Alpha from a nominal variable and 95% confidence intervals generated through bootstrapping with 1000 iterations (https://www.k-alpha.org/). Lower left side of matrix (orange) represents the percentage and number of children where an exact match between the two specified sources within the matrix was present.

Data on classification CC3 from at least one source were available for 3739 children, data from at least two sources were available for 2600 children. Where data from two or more sources were available, cleft classification matched across the available sources for 80.9% (n = 2104) of the sample. This resulted in a Krippendorf's Alpha of 0.818 (95% CIs 0.80, 0.84), suggesting a satisfactory level of agreement between sources. The two sources with the least agreement were the Cleft Multidisciplinary Collaborative and Cleft Collective Biological Sample Transfer Forms, with a 64.9% (n = 24) match in CC3 and a Krippendorf's Alpha of 0.562 (95% CIs 0.34, 0.73) suggesting inconsistent coding of the cleft classification [Table 3]. The two sources with the most agreement were CRANE and Medical Records, with a 96.6% (n = 425) match in CC3 and a Krippendorf's Alpha of 0.957 (95% CIs 0.94, 0.98) suggesting almost perfect agreement [Table 3]. A final CC3 cleft classification was determined for 3526 (94.3%) children and of those 67.7% (n = 2387) were validated across multiple sources.

**Table 3.** Matrix of Krippendorf's Alpha and Percentage Match for CC3 Cleft Classification Across Dual Sources.

Cleft Classification 3 R-UCL / L-UCL / BCL / CP / R-UCLP / L-UCLP / BCLP	CC Parental Questionnaires	CC Biological Sample Transfer Forms	CC Surgical Forms	Medical Records	Cleft Multidisciplinary Collaborative	CRANE
CC Parental questionnaires		0.764 (0.73, 0.80)	0.775 (0.74, 0.81)	0.664 (0.58, 0.75)	0.685 (0.58, 0.78)	0.626 (0.57, 0.68)
CC biological sample transfer forms	83.5 (n = 568)		0.845 (0.82, 0.87)	0.736 (0.67, 0.80)	0.562 (0.34, 0.73)	0.676 (0.63, 0.73)
CC Surgical forms	83.8 (n = 598)	88.3 (n = 1194)		0.870 (0.83, 0.91)	0.812 (0.74, 0.88)	0.817 (0.78, 0.85)
Medical records	73.3 (n = 129)	78.8 (n = 197)	89.6 (n = 326)		0.883 (0.83, 0.92)	0.957 (0.94, 0.98)
Cleft Multidisciplinary Collaborative	75.0 (n = 87)	64.9 (n = 24)	84.7 (n = 144)	90.5 (n = 229)		0.824 (0.78, 0.87)
CRANE	69.9 (n = 234)	74.0 (n = 367)	85.3 (n = 529)	96.6 (n = 425)	86.0 (n = 300)	

*CC3 represents the anatomical oral region, laterality and sidedness of the cleft: R-UCL – Right sided Unilateral Cleft Lip; L-UCL – Left sided Unilateral Cleft Lip; BCL – Bilateral Cleft Lip; CP – Cleft Palate only; R-UCLP – Right sided Unilateral Cleft Lip and Palate; L-UCLP – Left sided Unilateral Cleft Lip and Palate; BCLP – Bilateral Cleft Lip and Palate.

**CC - Cleft Collective; CRANE - The Cleft Registry and Audit NEtwork (CRANE) database.

***Upper right side of matrix (blue) represents Krippendorf's Alpha from a nominal variable and 95% confidence intervals generated through bootstrapping with 1000 iterations (https://www.k-alpha.org/). Lower left side of matrix (orange) represents the percentage and number of children where an exact match between the two specified sources within the matrix was present.

Data on classification LAHSAL from at least one source were available for 3427, and data from at least two sources were available for 1776 children. Where data from two or more sources were available, cleft classification matched across the available sources for 48.6% (n = 863) of the sample. This resulted in a Krippendorf's Alpha of 0.538 (95% CIs 0.52, 0.56), suggesting inconsistent coding between sources. The two sources with the least agreement were Cleft Multidisciplinary Collaborative and Cleft Collective short surgical forms, with a 37.2% match in LAHSAL and a Krippendorf's Alpha of 0.322 (95% CIs 0.26, 0.38) suggesting inconsistent coding [Table 4]. The two sources with the most agreement were medical records and CRANE, with a 78.4% match in LAHSAL and a Krippendorf's Alpha of 0.766 (95% CIs 0.73, 0.80) suggesting satisfactory agreement [Table 4]. A final LAHSAL cleft classification was determined for 2775 (81.0%) children and of those 40.5% (n = 1124) were validated across multiple sources.

**Table 4.** Matrix of Krippendorf's Alpha and Percentage Match for LAHSAL Cleft Classification Across Dual Sources.

Cleft Classification 4 LAHSAL	CC Surgical Forms	Medical Records	Cleft Multidisciplinary Collaborative	CRANE
CC Surgical forms		0.376 (0.33, 0.42)	0.322 (0.26, 0.38)	0.422 (0.39, 0.45)
Medical records	41.8 (n = 238)		0.628 (0.58, 0.67)	0.766 (0.73, 0.80)
Cleft Multidisciplinary Collaborative	37.2 (n = 106)	65.4 (n = 284)		0.626 (0.58, 0.67)
CRANE	46.1% (n = 439)	78.4 (n = 561)	65.5 (n = 376)	

*LAHSAL cleft classification denotes the anatomical location and complexity of an orofacial cleft, starting from right to left each letter represents an oral location where the cleft may present (right lip, right alveolus, hard palate, soft palate, left alveolus, left lip). Capital letters represent a complete cleft in that location, lowercase letters represent an incomplete cleft in that location and a dot or hyphen represents normal development.

**CC - Cleft Collective; CRANE - The Cleft Registry and Audit NEtwork (CRANE) database.

***Upper right side of matrix (blue) represents Krippendorf's Alpha from a nominal variable and 95% confidence intervals generated through bootstrapping with 1000 iterations (https://www.k-alpha.org/). Lower left side of matrix (orange) represents the percentage and number of children where an exact match between the two specified sources within the matrix was present.

Where LAHSAL data were available from more than two sources, inconsistencies in coding were mainly seen in the coding of the completeness of the cleft, accounting for 94.4% (n = 862) of inconsistencies. Inconsistencies in the reporting of sidedness were also present in 6.0% (n = 55) of cases. Inconsistencies in both completeness and sidedness were present in n = 18 of cases reported. The remaining 14 inconsistencies seen across LAHSAL classifications were inconsistencies in the affected orofacial structures or laterality of the cleft.

## Discussion

### Principal Findings

Our study aimed to assess the level of agreement for cleft classification within and between different raters and to validate cleft classification within the Cleft Collective. The simpler the cleft classification, the more agreement there was between sources. Once a cleft classification had more elements, such as laterality, sidedness and completeness, the more disagreement occurred.

Intra-rater agreement between maternal reports was almost perfect when reporting simpler cleft classification such as CC1 and CC2 with Krippendorf's Alpha values of 0.987 and 0.975, respectively. The addition of sidedness to the cleft classification, CC3, reduced the level of agreement to α=0.808. Very similar results were seen within paternal report of cleft classification with Krippendorf's Alpha values reported as α=0.980, α=0.963 and α=0.807 for CC1, CC2 and CC3 respectively. When asked about the sidedness of their child's cleft parents/guardians were asked, “when looking at your child, which side of the mouth is their cleft?”, this provides the opposite answer to the side in which the cleft occurs for the child and the side in which it is recorded in the classification. It is possible that this question may have been misread or misunderstood when reporting the side of the child's cleft. In addition, existing evidence suggests that approximately 14.6% of adults struggle to differentiate between left and right.^34^

Intra-rater agreement between Cleft Collective short surgical forms were reported as having almost perfect agreement or satisfactory agreement for cleft classifications CC1, CC2 and CC3. However, the level of agreement reduced when reporting LAHSAL for the same child at different timepoints with a Krippendorf's Alpha value of 0.672 suggesting inconsistent coding within the same rater.

When comparing cleft classifications CC1, CC2 and CC3 between parents, Krippendorf's Alpha values were all greater than 0.90 and suggested almost perfect agreement for CC1 and CC2 and satisfactory agreement for CC3. Across all seven sources, inter-rater agreement was almost perfect for CC1 with a Krippendorf's Alpha value of 0.957. Agreement was reduced to satisfactory for classifications CC2 and CC3 but reduced even further to inconsistent coding (α=0.538) when comparing LAHSAL classifications across the four sources that collected LAHSAL. When comparing agreement between two sources, agreement between the CMC and Cleft Collective biological transfer forms were consistently reported to have the lowest levels of agreement for CC1, CC2 and CC3. When exploring LAHSAL, the lowest agreement was seen between the CMC and Cleft Collective surgical forms. This could be because of differing interpretation of the classification or potentially due to the fact the responders to any of these sources may have had less training.

It is only by triangulating sources that we can have confidence in the labelling of phenotypes within large datasets. The use of data from seven different sources enabled us to determine the final classification of cleft phenotype for 4003 (98.8%) children using the simplest form of classification (CC1) and for 2775 (81.0%) for the most detailed classification (LAHSAL). A similar pattern of levels of agreement were seen across all sources for both intra- and inter-agreement with more detailed classification having less agreement.

### Consistency with Other Evidence

There is a paucity of literature on intra- and inter-rater agreement of cleft classification but McBride et al., using data from CleftSiS electronic records, showed higher levels of agreement within and between raters compared to our study.^8^ Across the fours raters used within the McBride study none of them had received official training on the use of the LAHSAL classification system but all four were given written instructions on how to classify a cleft using this system. Half of the raters also had extensive clinical experience within cleft care, but the other 50% of raters had no experience.^8^ It is unknown whether the raters within our study had received training on the LAHSAL classification, but all would have worked within cleft care when using LAHSAL to classify the cleft. The greater disagreement found within our study between raters may have been due to the raters recording the cleft classification during routine clinical care and not having dedicated time to work through instructions whilst classifying the cleft.

Houkes et al. tested the learnability of the LAHSAL classification system for 29 cleft care specialists. Learnability considers how easy it is for users to accomplish a task the first time they encounter the interface and how many repetitions it takes for them to become efficient at that task. The cleft care specialists were asked to classify a cleft using LAHSAL before receiving training and then asked to reclassify after watching a training webinar. Inter-rater agreement before receiving the training was extremely low with a Fleiss’ Kappa value of −0.015, suggesting more inconsistencies across raters than what would be expected by chance. Following training through the webinar, inter-rater agreement improved; however, the Fleiss’ Kappa value was still low (κ=0.217) and suggested inconsistencies between raters.^12^ Within our study, levels of inter-rater agreement were much higher than those reported within Houkes et al., yet inconsistencies were still present across raters.

Existing evidence suggests that training or the use of on-hand guidelines on the LAHSAL cleft classification system could be useful to ensure that cleft classification is recorded accurately. It is important for cleft classification to be recorded accurately for the purpose of clinical care and research, and care should be taken when recording the classification. Based on the findings with our study, it is recommended that LAHSAL classification should be validated by at least two members of the cleft team and a final validated classification should be reported within the medical records. The validation process might best be undertaken at the point of surgery when the child is undergoing their primary cleft repair.

## Strengths

We performed a national multi-centre study, comprising data from the whole of the UK and involving all centralised cleft-centres from the four nations. Data were obtained from seven sources to enable validation and the most accurate classification of a child's cleft. In previous studies sample sizes have been much smaller and/or have been from single centres.^8,12,14^ Our work was built on strong collaborations with the UK cleft teams, the CMC and CRANE. Where classifications differed with data provided by CRANE, the Cleft Collective was able to supply the validated classification back to CRANE to update their database.

## Limitations

There is a possibility that data recorded as being from different sources are in fact collected from the same location, i.e., the child's medical records or from the CRANE Database. When collecting data directly from medical records and through the CMC, where possible, data were purposively requested for those children where data were missing. This would have limited the numbers of children whose classification had been collected from the same source.

We cannot rule out the possibility that responders may have misread or misunderstood the question asked about sidedness within the parental questionnaires and the biological sample transfer forms. Given the inherent nature of classifying a cleft based on the side in which the cleft presents on the child there may be some confusion when asked to report which side the cleft appears when looking at the child. A correct response to this question would provide the opposite side to which the cleft actually appears, and the side reported within the cleft classification. Where a cleft classification has been validated across sources, inaccurate classifications of sidedness would have been phased out but this would still impact the percentage match and Krippendorf's Alpha.

## Clinical Importance

The validation of cleft classification is vital for several reasons within clinical practice, namely for treatment planning, outcome assessment, quality improvement, patient counselling and for research in cleft. Different types of orofacial cleft require different types of treatment, including surgical techniques and interventions. The validation of cleft classification ensures that patients receive the most effective treatment for their specific condition. Validated cleft classification facilitates the comparison of treatment outcomes across different cleft subtypes, patient demographics and healthcare settings. It enables clinicians to accurately assess the effectiveness of various treatment approaches and make informed decisions for future patient care. This validation study will help highlight the need for more accuracy within the reporting of the cleft classification, which in turn allows clinicians and healthcare providers to improve the quality of cleft care. Accurate classification of orofacial clefts enables healthcare providers to offer comprehensive counselling and education to patients and their families. Understanding the specific type and extent of the cleft can help families prepare for the treatment process, anticipate potential challenges, and set realistic expectations regarding outcomes. Finally, validated cleft classifications are essential for research purposes, enabling researchers to accurately categorise patients into the correct cleft sub-phenotype has a large impact on the reliability of research findings in the field of cleft care. This is important in evaluating treatment outcomes, identifying factors which may influence prognosis and ensuring correct classification in both genotype and phenotype studies.

## Future Work

To help validate remaining cleft classifications, further work could be undertaken using pre-operative photos and/or 3D images of the study child taken by medical photographers at cleft centres. Photographs and images are not available for all children but where they are this would provide further validation to the classifications only obtained from one source or where classifications mismatch between two sources. Further work could also be undertaken to develop specific training and guidelines to improve inter-rater agreement for LAHSAL.

## Conclusion

Our study found that there was greater agreement in cleft classification between sources when the complexity of the classification decreased. Differences were most apparent when reporting sidedness of unilateral cleft lip with or without palate and when reporting completeness of the cleft. Validation of cleft classification is important for both clinical care and research. Work conducted has resulted in us being able to determine a final cleft classification for between 81.0% and 98.8% of Cleft Collective study children, dependent on the classification used (CC1, CC2, CC3, LAHSAL). Despite inconsistencies seen across sources, the use of LAHSAL is still advocated given its detailed nature of describing the cleft phenotype. It is recommended that all team members within the cleft team should have full training on the use of the LAHSAL classification and the classification should be checked and confirmed at the point of surgical repair of the cleft.

## Data Availability

The data that support the findings of this study are available on request directly from the Cleft Collective. Details on how to access the Cleft Collective resource can be found at http://www.bristol.ac.uk/cleft-collective/professionals/access/. Data are not publicly available due to privacy or ethical restrictions.
